# Enhanced bone exposure via laparoscopy in acetabulum and pelvic ring surgeries

**DOI:** 10.1007/s00264-025-06515-2

**Published:** 2025-03-31

**Authors:** Guillaume David, Marine Giorgi, Florian Bernard, Remi Di Francia, Cyril Mauffrey, Louis Rony

**Affiliations:** 1 Department of Bone Surgery, CHU Angers, 4 rue Larrey, Angers, 49100 France; 2 Laboratoire d’Anatomie, Faculté de Médecine, rue Haute de Reculée, Angers, 49045 France; 3 Denver Health Medical Center, Department of Orthopedics, 777 Bannock Street, Denver, CO 80204 USA; 4 CHU Brest Cavale Blanche, Boulevard Tanguy Prigent, Brest, 29200 France

**Keywords:** Acetabular fractures, Pelvic endoscopy, Anterior intrapelvic approach, Modified, Stoppa approach, Anatomy of the acetabulum

## Abstract

**Purpose:**

In orthopaedic surgery, achieving optimal exposure for acetabular and pelvic ring fractures with minimal invasiveness remains a challenge. This study compares bone exposure in key pelvic zones using an endoscopic approach versus the AIP (Modified Stoppa) in cadaveric specimens.

**Materials and methods:**

We dissected ten adult cadaveric bodies, obtained from our institution’s body donation program, using an extraperitoneal endoscopic dissection on one side and an AIP approach on the other. Bone areas were marked at each step of dissection by drill holes to measure the bone exposure surface for each zone (true and false pelvis) between the laparoscopic and open approaches. A Student’s t test was used to compare the exposure areas obtained.

**Results:**

The average age of the cadavers was 83 years, with a balanced representation of genders (60% male, 40% female). Comparison of zones between endoscopy and AIP found for Zone 1: 1.4 cm^2^ (range − 3.813 to 1.013) for AIP with no statistical significance. For Zone 2: 0.5 cm^2^ (range − 1.9141 to 2.9141) for AIP with no statistical significance. For Zone 3: 0,6 cm^2^ (range − 1.0243 to 2.2243) for AIP with no statistical significance. And for Zone 4: 3.5 cm^2^ (1.874; 5.126) for endoscopy with statistical significance (*p* = 0.001).

**Conclusion:**

Our study demonstrates that the endoscopic method provides comparable visualization of the different pelvic zones compared to the open method (AIP), with enhanced access to Zone 4, a crucial area in managing acetabulum and pelvic ring fractures.

**Level of evidence:**

Level V, cadaveric study.

**Supplementary Information:**

The online version contains supplementary material available at 10.1007/s00264-025-06515-2.

## Introduction

The anterior intrapelvic extraperitoneal (AIP) approach has revolutionized the internal fixation of acetabular fractures, establishing itself as a cornerstone in the field of orthopaedic surgery. Initially introduced by Stoppa for hernia repairs [1–3], and subsequently adapted for pelvic bone surgery by Hirvensalo [4], the technique has undergone further modifications by Cole [3], enhancing its applicability and effectiveness. This evolution has seen the AIP approach supplant the traditional ilio-inguinal method for a majority of acetabular fractures. Extensive literature has documented the surgical technique, the anatomical landmarks encountered [5], and the myriad advantages it offers. Among its most celebrated benefits is the simultaneous access it provides to both columns of the acetabulum [6], facilitating the management of complex fracture patterns, particularly those involving medial displacement of the acetabular columns or the quadrilateral surface [6, 7].

The modified Stoppa approach’s utility in offering unparalleled visualization below the arcuate line of the coxal bone has been a game-changer, simplifying the placement of reduction clamps [8] and enhancing surgical outcomes [9]. Studies, including those by Bible et al., have quantified the extent of bony pelvis exposure achievable through this approach, highlighting its capacity to expose critical areas from the pubic symphysis to the sacroiliac joint [10]. Furthermore, recent advancements have seen the introduction of endoscopic techniques to pelvic surgery, promising even greater bone exposure [11]. Our team’s pioneering work in this area revealing that endoscopic bone exposure potentially surpasses that of the AIP approach, albeit without a detailed comparative analysis of the specific areas exposed. We recently proposed a zone system creating a map to measure the different zones of the innominate bone more precisely [12].

The aim of our study is to address this knowledge gap by comparing bone exposure between the endoscopic and AIP approaches across different pelvic regions. This analysis is important as it may influence surgical decision-making and the adoption of techniques that improve outcomes in acetabular fracture management. By quantifying and comparing bone exposure for each approach, our study seeks to provide relevant data to guide surgical practice in pelvic trauma.

## Materials and methods

### Dissection protocol

In our study, we examined ten adult cadaveric bodies, graciously donated to our institution’s body donation program (ethical approval registration number IRB: 492201). Each specimen was preserved using a 10% formalin solution, ensuring the integrity of the tissues for dissection. We recorded the age and sex of each specimen.

**Fig. 1:**
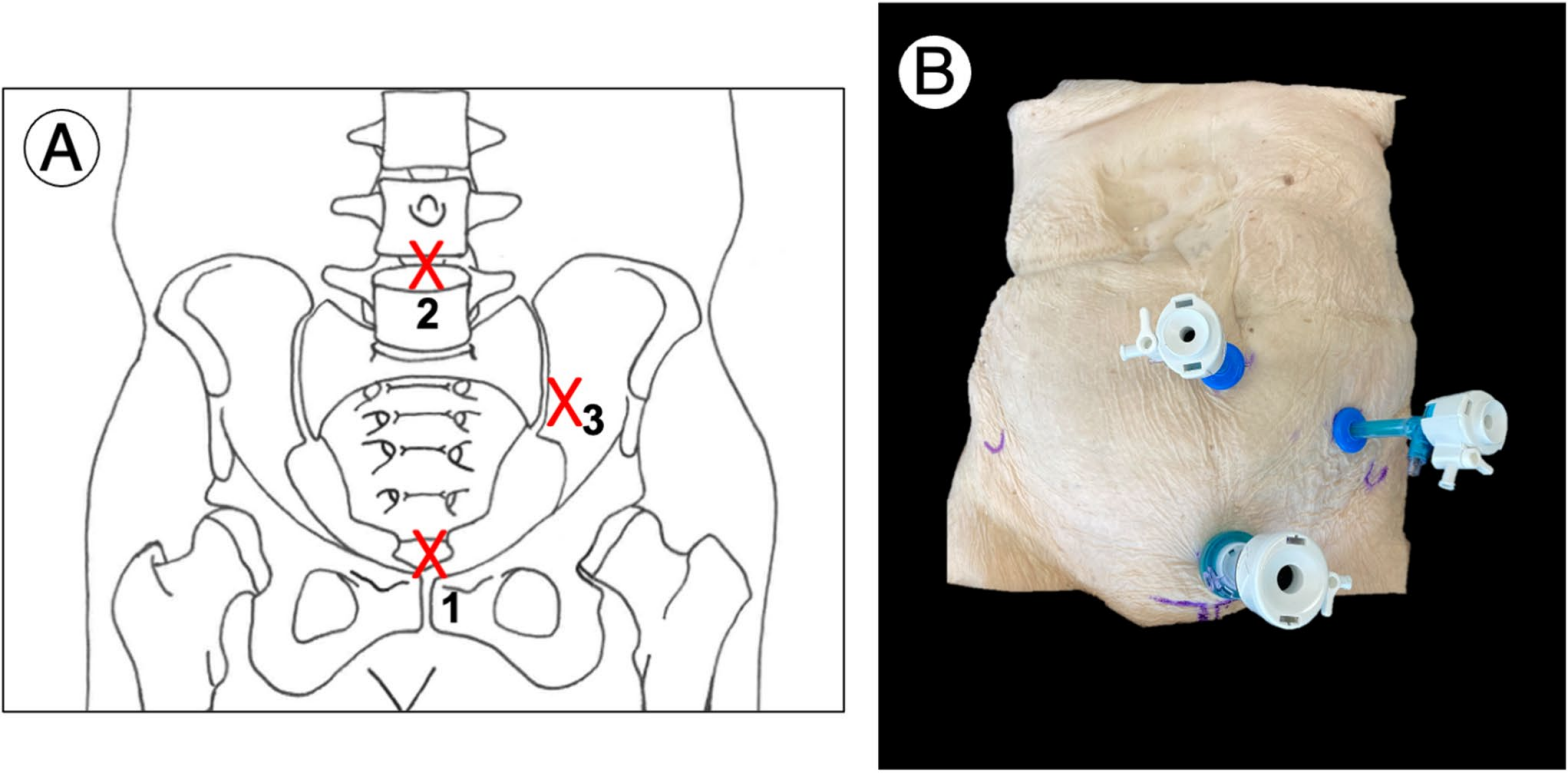
Diagram illustrating the placement of the trocars (**A**). Photograph showing the setup of the trocars (**B**). Dissection performed on the left side

The dissection process was initiated with an extraperitoneal endoscopic approach, adhering to a previously established protocol. The initial phase involved the placement of laparoscopic trocars: a 12-mm trocar was inserted 2 cm above the pubic symphysis along the median line, a 15-mm trocar was positioned 4 cm below the umbilicus, and a third 5-mm trocar was placed in the para rectus space, located at the external third of the line stretching from the antero-superior iliac spine to the umbilicus, this section is shown Fig. 1. The dissection extended from the symphysis to the sacroiliac (SI) joint, utilizing anatomical landmarks within the Pelvic Zone as guides. Bone areas were marked with 3.2 mm drill holes to delineate the limits of exposure.

Following the initial phase, an Anterior Intra Pelvic approach (AIP) was employed for the second part of the dissection, using the same method to mark bone areas.

### Demographics and description of study population

The study documented the demographic details of the cadaveric specimens. This included age and sex distribution, contextualizing the findings within broader anatomical and clinical discussions.

### Data and measurement

The characteristics of the subjects, including age, gender, and the side of endoscopic dissection, were documented. A single surgeon, with extensive training in pelvic and acetabular surgery, performed all dissections to ensure consistency and accuracy. Following the dissection, soft tissues were removed from the osseous pelvis, and each pelvis was scanned using an EinScan Pro HD (SHINING 3D^®^ Tech Co., Ltd). This advanced portable scanner employs photogrammetry technology to facilitate 3D reconstruction and surface measurements, allowing for evaluation of the drilled holes outlining the exposed bone area.

**Fig. 2:**
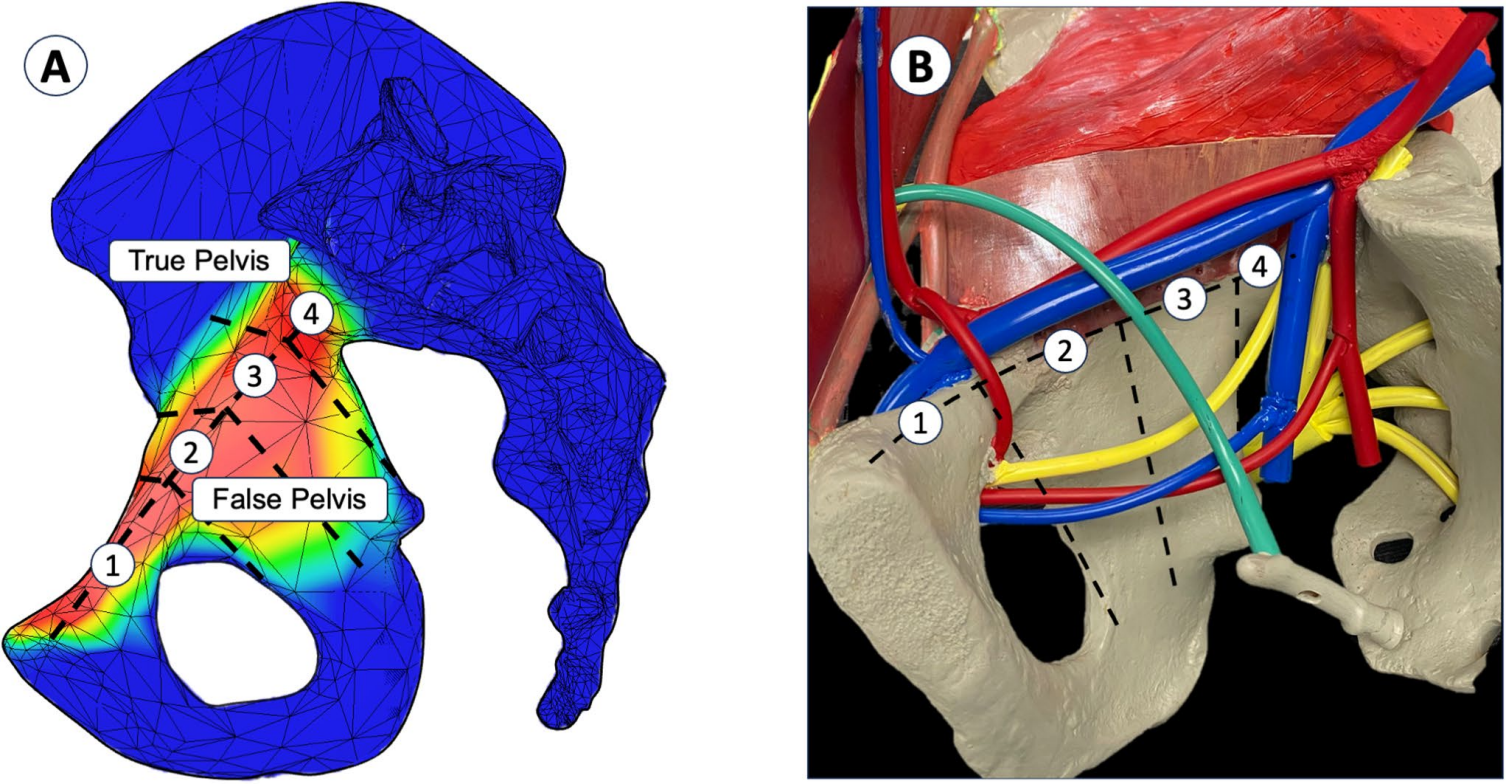
Endopelvic zone segmentation (**A**); Model representing the useful anatomical structures (**B**)

For a detailed comparison of bone surface exposure, the coxal bone was divided into four endopelvic zones of clinical interest, each further subdivided in relation to the arcuate line into true and false pelvis zones [11]. To sum up, Zone 1 extends from the symphysis to the iliopectineal eminence, with the corona mortis at the limit. Zone 2 lies between the corona mortis and the middle of the quadrilateral surface, in front of the vas deferens or the round ligament and above the obturator pedicle. Zone 3 lies behind zone 2 and in front of the greater sciatic notch. Zone 4 extends from the greater sciatic notch to the sacral alar. The upper limit of the zones is the external iliac vessels. Figure 2 shows these zones and their anatomical environments. This segmentation facilitated a nuanced analysis of exposure surfaces across clinically relevant areas.

### Statistical analysis

Statistical analysis was conducted using R software, version 3.6.1 (R Foundation for Statistical Computing). Qualitative variables were expressed in numbers and percentages, while quantitative variables were presented as mean and standard deviation (SD). The independence between quantitative variables was assessed using Student’s test, with a significance threshold set at 5%. Confidence intervals were calculated at 95%.

## Results

### Patient demographics

In this study, we analyzed ten subjects, achieving a balanced representation of genders with 60% male (*n* = 6) and 40% female (*n* = 4) specimens. The subjects have an age range from 70 to 97 years, with an average age of 83 years. These demographics are summarized in Table 1.

**Table 1:** Subject and dissection exposure data

													Mean +/- SD		
Cases:		1	2	3		4	5	6	7	8	9	10	-		
	Gender*	F	M	M		M	F	F	F	M	M	M		-	
	Age	94	83	78		87	70	82	97	81	80	78	83 ± 8 (70–97)		
Bone Exposure (cm^2^):													Bone Exposure	True pelvis	False pelvis
	Endoscopy														
	Zone 1 (TP/FP)	10 (8/2)	11 (5/6)	15 (7/8)		13 (7/6)	11 (7/4)	18 (10/8)	15 (8/7)	21 (13/8)	22 (13/9)	24 (13/9)	16 ± 5 (10–24)	9 ± 3 (5–13)	8 ± 2 (2–9)
	Zone 2 (TP/FP)	21 (7/14)	23 (13/10)	24 (14/10)		25 (15/10)	20 (12/8)	19 (9/10)	19 (10/9)	25 (16/9)	26 (17/9)	24 (16/8)	23 ± 3 (19–26)	13 ± 3 (7–17)	10 ± 2 (8–14)
	Zone 3 (TP/FP)	16 (4/12)	18 (10/8)	20 (13/7)		16 (9/6)	16 (9/7)	15 (9/6)	19(12/7)	29 (20/9)	28 (19/9)	26 (16/10)	20 ± 5 (15–29)	12 ± 5 (4–20)	8 ± 2 (6–12)
	Zone 4 (TP/FP)	14 (6/8)	15 (7/8)	15 (8/7)		12 (6/6)	12 (7/5)	12 (7/5)	19 (11/8)	19 (13/6)	17 (10/7)	18 (11/7)	15 ± 3 (12–19)	9 ± 2 (6–13)	7 ± 1 (5–8)
	Total (TP/FP)	61(25/36)	67 (40/37)	74 (42/32)		66 (37/28)	59 (35/24)	64 (35/29)	72 (41/31)	94 (62/32)	93 (59/34)	92 (56/34)	74 ± 14 (59–94)	43 ± 12 (25–62)	31 ± 4 (24–37)
	Modified Stoppa														
	Zone 1 (TP/FP)	16 (7/9)	17 (9/8)	17 (8/9)		14 (8/6)	13 (4/9)	12 (7/5)	15 (9/6)	21 (9/12)	24 (13/11)	25 (10/15)	17 ± 5 (12–25)	8 ± 2 (4–13)	9 ± 3 (5–15)
	Zone 2 (TP/FP)	23 (11/12)	19 (12/7)	27 (15/12)		23 (11/12)	16 (7/9)	13 (7/6)	22 (13/9)	28 (16/12)	25 (17/8)	25 (11/14)	22 ± 5 (13–28)	12 ± 3 (7–17)	10 ± 3 (6–14)
	Zone 3 (TP/FP)	12 (7/5)	18 (11/7)	16 (8/8)		17 (10/7)	14 (6/8)	17 (12/5)	21 (12/9)	29 (1712)	26 (17/9)	27 (19/8)	20 ± 6 (12–29)	12 ± 5 (6–19)	8 ± 2 (5–12)
	Zone 4 (TP/FP)	12 (6/6)	11 (6/5)	13 (7/6)		11 (7/4)	5 (4/1)	10 (6/4)	15 (7/8)	12 (6/6)	12 (6/6)	17 (10/7)	12 ± 3 (5–17)	7 ± 2 (4–10)	5 ± 2 (1–8)
	Total (TP/FP)	63 (31/32)	65 (38/27)	73 (38/35)		65 (36/29)	48 (21/27)	52 (32/20)	73 (41/32)	90 (48/42)	87 (53/56)	94 (50/44)	71 ± 16 (48–94)	39 ± 10 (21–53)	34 ± 10 (20–56)
Dissection completed		Yes	Yes	Yes		Yes	Yes	Yes	Yes	Yes	Yes	Yes	-	-	-

*Sex ratio: 1.5; TP: True Pelvis; FP: False Pelvis

### Bone exposure surface analysis

**Fig. 3:**
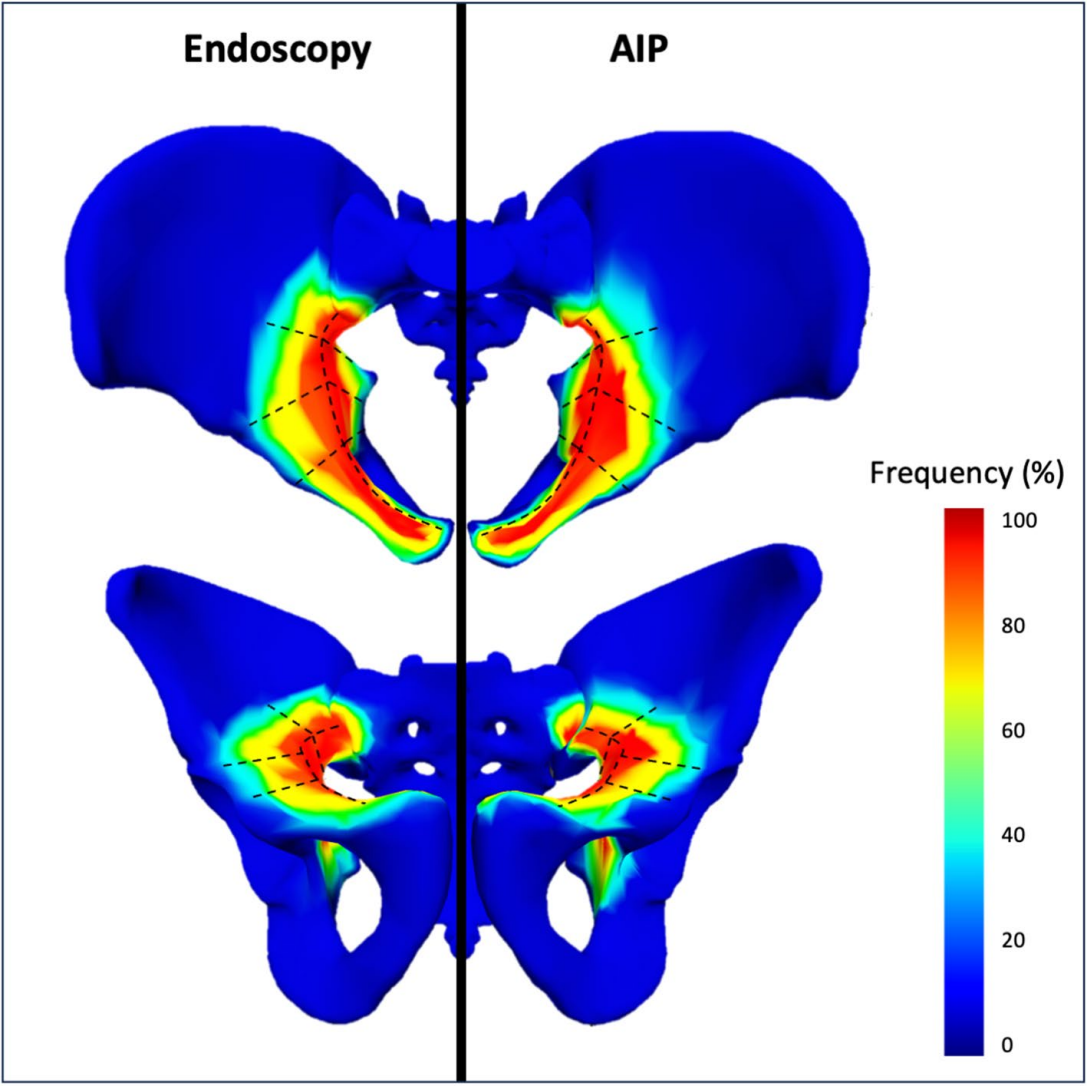
Heatmaps of exposure surfaces between Endoscopic and AIP approaches, the most frequently exposed areas in red and the least exposed areas in blue

**Fig. 4:**
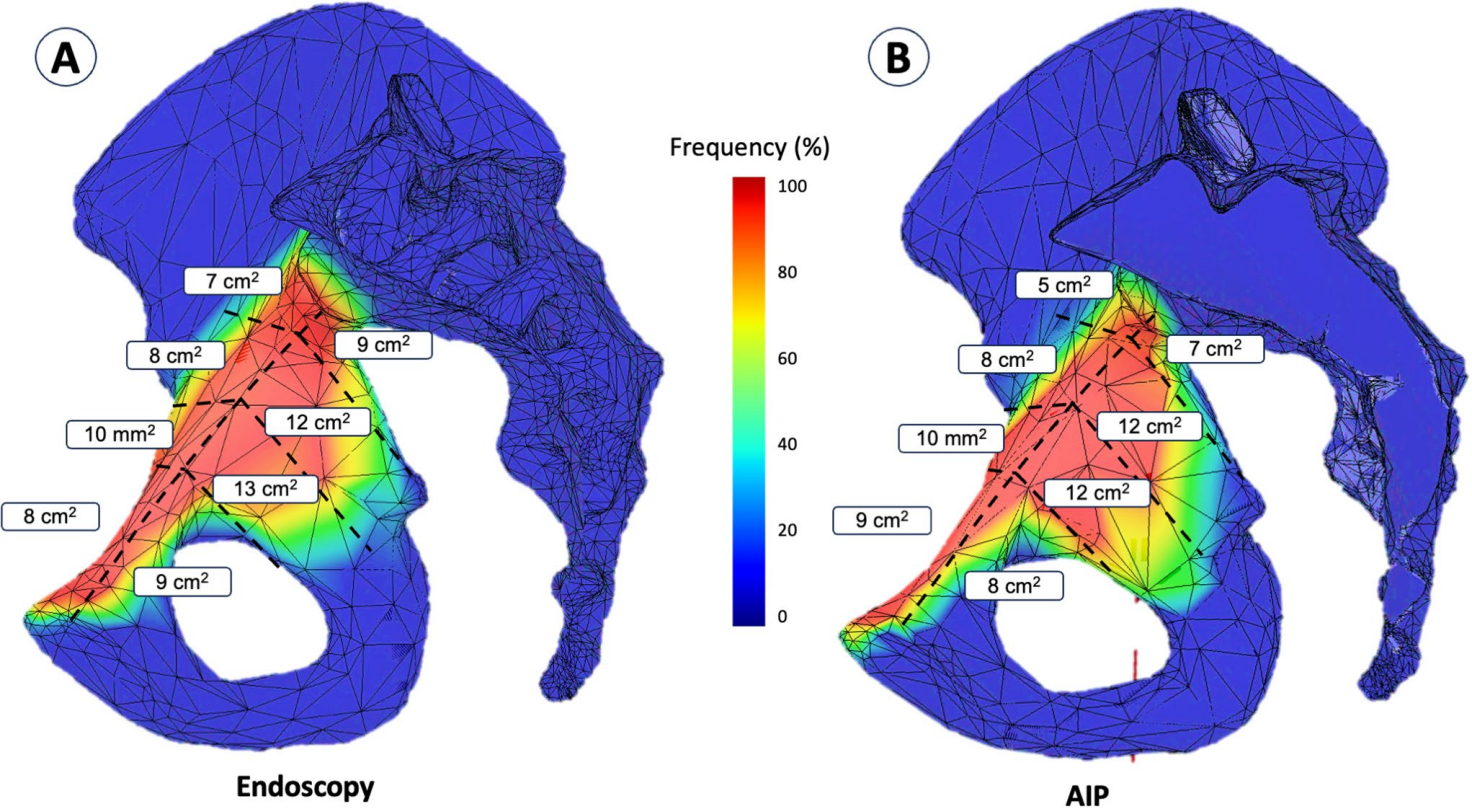
Distribution of bones exposure areas for each zone for the Endoscopic (**A**) and AIP (**B**) approach

Through endoscopic exposure, we observed for each zones: Zone 1 revealed an exposure of 16 cm² ± 5 (10 to 24), Zone 2 showed 23 cm² ± 3 (19 to 26), Zone 3 had 20 cm² ± 5 (15 to 29), and Zone 4 presented 15 cm² ± 3 (12 to 19). Conversely, the Modified Stoppa exposure technique offered: Zone 1 at 17 cm² ± 5 (12 to 25), Zone 2 at 22 cm² ± 5 (13 to 28), Zone 3 at 20 cm² ± 6 (12 to 29), and Zone 4 at 12 cm² ± 3 (5 to 17). These detailed findings are depicted in Table 1 and visually represented through heat maps in Figs. 3 and 4. The heatmap provides a comprehensive overview of all dissections performed for an approach, highlighting the most frequently exposed areas in red and the least exposed areas in blue.

### Comparative analysis of zones surfaces

Upon comparing the bone exposure surfaces between endoscopic and AIP techniques, we found for Zones 1: 1.4 (-3.813; 1.013) for AIP with no statistical significance (*p* = 0,2), for Zone 2: 0.5 (-1.9141; 2.9141) for AIP with no statistical significance (*p* = 0,7), and for Zone 3: 0,6 (-1.0243; 2.2243) for AIP with no statistical significance (*p* = 0,4). However, for Zone 4 endoscopy achieved better exposure: 3.5 (1.874; 5.126) with a statistical significance (*p* = 0.001). When comparing the total bone exposure of the true and false pelvis, endoscopy provide 43 cm² ± 12 (25 to 62) exposure of the true pelvis, versus 39 cm² ± 10 (21 to 53 cm²) for the AIP, with no statistical significance (*p* = 0.05). These detailed results are presented in Table 2.

**Table 2:** Dissection exposure between endoscopy and modified Stoppa

Approache	Endoscopy	Modified Stoppa		Mean Difference*	*P Value*
**Zone’s Bone Exposure** (cm^2^)					
Zone 1	16 ± 5 (10–24)	17 ± 5 (12–25)		1.4 (-3.813; 1.013)	0.2
Zone 2	23 ± 3 (19–26)	22 ± 5 (13–28)		0.5 (-1.9141; 2.9141)	0.7
Zone 3	20 ± 5 (15–29)	20 ± 6 (12–29)		0,6 (-1.0243 ; 2.2243)	0.4
Zone 4	15 ± 3 (12–19)	12 ± 3 (5–17)		3.5 (1.874 ; 5.126)	*0.001*
Total Bone Exposure	74 ± 14 (59–94)	71 ± 16 (48–94)		3.2 (-0.4132 ; 6.8132)	0.07
True Pelvis Bone Exposure	43 ± 12 (25–62)	39 ± 10 (21–53)		4.4 (0.0265 ; 8.7735)	0.05
False Pelvis Bone Exposure	31 ± 4 (24–37)	34 ± 10 (20–56)		2.7 (-9.5619 ; 4.1619)	0.4

*The values are given as the mean (95% confidence interval)

## Discussion

This study demonstrates that the endoscopic approach is comparable to the AIP approach in terms of bone exposure surface across most zones, while providing superior exposure in zone 4. This Zones nomenclature could be an interesting instrument when deciding between endoscopy and the Anterior Intrapelvic (AIP) method for pelvic examinations. Our findings reveal that for zones 1, 2, and 3, both methods are equally effective. However, the distinction becomes apparent in zone 4, where endoscopy demonstrates superiority. This zone, crucial for the management of acetabulum and pelvic ring fractures [13], corresponds anatomically to the pillar of the innominate bone [14], a region of paramount importance due to its role in the convergence of fractures [12] and its high-quality bone tissue [15].

Comparing our results with those in existing literature, such as the studies by Yin et al. [16] and Sagi et al. [2, 6], reinforces the significance of zone 4 in the context of both-column acetabular fracture mapping and the reduction of posterior column fractures. These studies highlight the critical nature of this zone in surgical planning and execution, aligning with our findings on the advantages of endoscopic exploration in this area. Furthermore, our research suggests that laparoscopy may offer superior exploration of the true pelvis, essential for treating acetabular fractures involving the posterior column. This advantage is particularly notable when considering the access it provides to both supra and infra-arcuate line structures, enhancing the surgeon’s ability to manage anatomical structures more effectively than the AIP approach.

Despite the promising implications of our study, it is not without limitations. The primary constraint is the reliance on endoscopic dissection on embalmed anatomic specimens, which may not accurately represent the conditions in living subjects due to tissue retraction and space constraints. Additionally, the average age of our subjects was 83 years, indicating a focus on a demographic that, while likely to benefit most from minimally invasive techniques, may not encompass the full spectrum of patients requiring pelvic surgery. These limitations notwithstanding, our step-by-step methodological approach has laid the groundwork for future research aimed at exploring the feasibility of reducing and fixing acetabular and pelvic ring injuries through minimally invasive techniques.

Our study contributes valuable insights into the comparative effectiveness of endoscopy and the AIP method in pelvic exploration, with a particular focus on the critical zone 4. By highlighting the anatomical and surgical relevance of this zone, our research supports the continued development and application of endoscopic techniques in the treatment of pelvic fractures, promising improved outcomes for a demographic characterized by constitutional fragility.

## Conclusions

The findings underscore the efficacy of the endoscopic method in offering comparable visualization of various pelvic zones to that of the traditional open method. Significantly, it demonstrates superior access to zone 4, a critical area in managing acetabulum and pelvic ring fractures. These insights suggest that the endoscopic approach could potentially revolutionize the treatment protocols for such injuries by providing enhanced access to crucial areas. However, to fully ascertain the viability and benefits of employing the endoscopic method for the reduction and fixation of acetabular fractures and pelvic ring injuries, further in-depth feasibility studies are imperative. Our research paves the way for future investigations aimed at refining surgical techniques, ultimately improving patient outcomes in the treatment of complex pelvic injuries.

## Electronic supplementary material

Below is the link to the electronic supplementary material.

Supplementary Material 1

Supplementary Material 2

Supplementary Material 3

Supplementary Material 4

Supplementary Material 5

Supplementary Material 6

## Data Availability

The authors make the data freely available.
